# Editorial: Smart forecasting: deep learning and explainable AI for real-world time series prediction

**DOI:** 10.3389/fdata.2026.1954626

**Published:** 2026-09-10

**Authors:** Leonard Barolli, Antonino Ferraro, Antonio Galli, Francesco Moscato

**Affiliations:** 1 Department of Information and Communication Engineering, Fukuoka Institute of Technology, Fukuoka, Japan; 2 Department of Information Science and Technology, Pegaso University, Naples, Italy; 3 Department of Electrical Engineering and Information Technology, University of Naples Federico II, Naples, Italy; 4 Department of Information and Electrical Engineering and Applied Mathematics, University of Salerno, Fisciano, Italy

**Keywords:** decision support systems, explainable artificial intelligence (XAI), hybrid forecasting models, large language models (LLMs), predictive analytics, time series forecasting

Artificial intelligence (AI) is increasingly transforming forecasting and predictive analytics across a wide range of scientific and industrial domains (Colace et al., 2026; Mondal et al., 2025). While traditional forecasting has long relied on statistical and econometric methods, recent advances in machine learning (ML), deep learning (DL), explainable artificial intelligence (XAI), and, more recently, large language models (LLMs) have substantially expanded the capability of predictive systems to model complex temporal dynamics, integrate heterogeneous information sources, and support data-driven decision-making (Jin and Ma, 2026; Ferraro et al., 2025). Rather than focusing exclusively on improving predictive accuracy, current research is progressively emphasizing forecasting systems that are interpretable, adaptive, and 10 capable of assisting human decision-makers in increasingly complex environments (Li and Tian, 2026; Amato et al., 2025).

This evolution has been fueled by the growing availability of multimodal data, advances in computational intelligence, and the widespread adoption of AI in high-impact domains such as healthcare, finance, software engineering, and industrial systems (Bhushan and Misra, 2025; Giudici and Wu, 2025; Siala and Lano, 2025; Ferraro et al., 2026). Modern forecasting models increasingly combine structured time-series data with textual information, graph representations, contextual knowledge, and domain expertise, enabling richer representations of complex phenomena (Kim et al., 2025). At the same time, the need for trustworthy AI has highlighted the importance of explainability and transparency, particularly in application scenarios where forecasting outcomes directly influence operational or strategic decisions (Gravina et al., 2026; Kalasampath et al., 2025).

Against this backdrop, this Research Topic, *Smart forecasting: deep learning and explainable AI for real-world time series prediction*, brings together six contributions that collectively illustrate recent advances in intelligent forecasting methodologies. Although the accepted articles investigate different application domains, they share the common objective of developing AI-driven forecasting systems that combine predictive performance with explainability and practical decision support.

For analytical clarity, the contributions can be organized into four interrelated thematic areas: Smart forecasting beyond finance; deep learning and hybrid models for financial forecasting; large language models for next-generation forecasting; and perspectives on intelligent forecasting. Together, these themes reflect the growing maturity of AI-based forecasting research and its evolution toward increasingly explainable, hybrid, and domain-aware predictive systems.

• *Smart forecasting beyond finance*. Forecasting techniques are increasingly expanding beyond their traditional applications in economics and finance, addressing operational challenges in domains where anticipating future events can significantly improve decision-making.

Leema et al. propose HairSentinel, a time-aware anomaly detection framework for forecasting hairfall trends using Temporal Fusion Transformers. Instead of relying on image-based diagnosis, the proposed approach models longitudinal behavioral and physiological information collected through user interactions, enabling early identification of abnormal hairfall patterns while maintaining an accessible and computationally efficient data collection process. The study illustrates how deep learning can support preventive healthcare by combining temporal modeling with anomaly detection.

Similarly, Aversano et al. investigate forecasting bug resolution times in open-source software projects through a comparative analysis of classical machine learning and deep learning approaches under both local and global forecasting scenarios. Besides evaluating forecasting performance cross multiple repositories, the study integrates explainable AI techniques—including permutation importance, saliency maps, and embedding analysis—to identify the software process characteristics that most strongly influence prediction outcomes.

Together, these studies suggest that intelligent forecasting is becoming an increasingly valuable decision-support technology beyond conventional financial applications. They demonstrate how combining deep learning with explainability enables more transparent and actionable predictions in domains where operational planning, resource allocation, and early intervention play a central role.

• *Deep learning and hybrid models for financial forecasting*. Financial forecasting remains one of the most dynamic research areas for artificial intelligence, with recent studies increasingly exploring hybrid approaches that combine statistical modeling with modern AI techniques.

Alnajjar et al. examine the determinants of banking stability within the Saudi financial system 54 through an integrated framework that combines econometric analysis with ARIMA and exponential 55 smoothing models. Their findings indicate that capitalization, profitability, asset quality, and bank 56 size significantly influence financial stability, while long-term forecasting provides useful support for 57 banking supervision and policy planning within the Saudi Vision 2030 framework.

Complementing this perspective, AL-Besher and AL-Najjar compare traditional GARCH-family models with Long Short-Term Memory (LSTM) neural networks for forecasting returns and volatility in the Saudi stock market. Their results show that GARCH models remain particularly effective for return prediction, whereas LSTM networks better capture nonlinear volatility dynamics, highlighting the complementary strengths of econometric and deep learning approaches for financial risk management and investment decision-making.

Collectively, these contributions indicate that contemporary financial forecasting is progressively moving toward hybrid predictive frameworks. Rather than replacing classical statistical models, deep learning increasingly complements established econometric techniques by capturing nonlinear temporal dependencies while preserving the interpretability required for financial decision-making.

• *Large language models for next-generation forecasting*. The emergence of large language models is opening new research directions for intelligent forecasting by enabling the integration of numerical and textual information within unified predictive frameworks.

Wang and Yeung propose an innovative LLM-driven forecasting framework for stock information networks that combines financial time series, institutional fund disclosures, retrieval-augmented generation, and graph representations. By jointly reasoning over structured numerical indicators and unstructured textual information, the proposed methodology significantly improves forecasting accuracy while providing economically interpretable explanations of evolving market dynamics.

This contribution illustrates a broader transition in forecasting research, where language models are progressively evolving from natural language processing tools into general reasoning systems capable of supporting complex predictive tasks. Their ability to integrate heterogeneous information sources appears particularly promising for forecasting applications characterized by dynamic, multimodal, and interconnected data.

• *Perspectives on Intelligent Forecasting*. Besides methodological advances, forecasting research also benefits from comprehensive analyses that consolidate current knowledge and identify future research opportunities.

Rohan et al. present a comprehensive review of artificial intelligence for financial market prediction, discussing the evolution from traditional statistical approaches toward machine learning, deep learning, reinforcement learning, blockchain-enabled financial systems, and quantum computing. Beyond summarizing existing forecasting methodologies, the review examines challenges related to explainability, ethical AI, data quality, regulatory compliance, and hybrid intelligence, while outlining future directions for intelligent financial decision support.

Viewed within the broader context of this Research Topic, this review provides an important conceptual framework that connects the methodological advances presented by the empirical studies. It highlights how forecasting research is progressively converging toward integrated AI ecosystems where predictive performance, transparency, adaptability, and human-centered decision support become equally important research objectives.

Although the accepted contributions span diverse application domains, several common research trends emerge across this Research Topic.

First, forecasting is progressively evolving from purely numerical prediction toward intelligent decision-support systems capable of integrating heterogeneous information, including structured time series, textual data, graph representations, and domain knowledge. This richer representation enables forecasting models to better capture the complexity of real-world phenomena and provide more informative predictions.

Second, explainability is increasingly becoming a fundamental design requirement rather than an optional feature. Several contributions explicitly incorporate explainable AI techniques to improve model transparency, facilitate interpretation, and increase users' trust in AI-assisted predictions. As forecasting systems become more deeply integrated into operational workflows, explainability will likely play a key role in their widespread adoption.

Third, the collected studies consistently highlight the value of hybrid intelligence. Rather than viewing statistical methods and AI-based approaches as competing paradigms, they demonstrate that combining econometric models, machine learning, deep learning, graph analytics, and large language models often leads to more robust, accurate, and interpretable forecasting systems. Likewise, the emergence of foundation models suggests that future forecasting frameworks will increasingly combine reasoning capabilities with predictive modeling, enabling AI systems to process heterogeneous information while generating more meaningful explanations for human decision-makers.

Overall, the contributions collected in this Research Topic demonstrate that smart forecasting is entering a new stage characterized by increasingly intelligent, explainable, and application-oriented predictive systems. Across healthcare, software engineering, banking, and financial markets, the studies consistently show that forecasting performance alone is no longer sufficient. Instead, practical adoption increasingly depends on the ability of AI systems to provide transparent, robust, and actionable predictions that effectively support human decision-making.

The diversity of the methodologies presented—including econometric modeling, machine learning, deep learning, explainable AI, graph-based analytics, and large language models—reflects the multidisciplinary nature of contemporary forecasting research and highlights the growing convergence of these paradigms into hybrid forecasting frameworks. Rather than replacing existing methodologies, AI is progressively extending them, enabling richer representations of temporal phenomena and more effective decision support across a wide variety of real-world applications.

We hope that this Research Topic will stimulate further interdisciplinary research on trustworthy AI-driven forecasting systems that combine predictive accuracy with explainability, adaptability, and human-centered design. As forecasting continues to evolve alongside advances in artificial intelligence, these characteristics will likely become fundamental for the next generation of intelligent predictive systems.
